# The *Biomphalaria glabrata* DNA methylation machinery displays spatial tissue expression, is differentially active in distinct snail populations and is modulated by interactions with *Schistosoma mansoni*

**DOI:** 10.1371/journal.pntd.0005246

**Published:** 2017-05-16

**Authors:** Kathrin K. Geyer, Umar H. Niazi, David Duval, Céline Cosseau, Chad Tomlinson, Iain W. Chalmers, Martin T. Swain, David J. Cutress, Utibe Bickham-Wright, Sabrina E. Munshi, Christoph Grunau, Timothy P. Yoshino, Karl F. Hoffmann

**Affiliations:** 1 Institute of Biological, Environmental and Rural Sciences, Aberystwyth University, Penglais Campus, Aberystwyth, United Kingodm; 2 Université Perpignan Via Domitia, CNRS, IFREMER, Perpignan, France; 3 Genome Sequencing Center, Washington University School of Medicine, St. Louis, Missouri, United States of America; 4 Department of Pathobiological Sciences, School of Veterinary Medicine University of Wisconsin, Madison, United States of America; Fundaçao Oswaldo Cruz, BRAZIL

## Abstract

**Background:**

The debilitating human disease schistosomiasis is caused by infection with schistosome parasites that maintain a complex lifecycle alternating between definitive (human) and intermediate (snail) hosts. While much is known about how the definitive host responds to schistosome infection, there is comparably less information available describing the snail’s response to infection.

**Methodology/Principle findings:**

Here, using information recently revealed by sequencing of the *Biomphalaria glabrata* intermediate host genome, we provide evidence that the predicted core snail DNA methylation machinery components are associated with both intra-species reproduction processes and inter-species interactions. Firstly, methyl-CpG binding domain protein (*Bgmbd2/3*) and DNA methyltransferase 1 (*Bgdnmt1*) genes are transcriptionally enriched in gonadal compared to somatic tissues with 5-azacytidine (5-AzaC) treatment significantly inhibiting oviposition. Secondly, elevated levels of 5-methyl cytosine (5mC), DNA methyltransferase activity and 5mC binding in pigmented hybrid- compared to inbred (NMRI)- *B*. *glabrata* populations indicate a role for the snail’s DNA methylation machinery in maintaining hybrid vigour or heterosis. Thirdly, locus-specific detection of 5mC by bisulfite (BS)-PCR revealed 5mC within an exonic region of a housekeeping protein-coding gene (*Bg14-3-3*), supporting previous *in silico* predictions and whole genome BS-Seq analysis of this species’ genome. Finally, we provide preliminary evidence for parasite-mediated host epigenetic reprogramming in the schistosome/snail system, as demonstrated by the increase in *Bgdnmt1* and *Bgmbd2/3* transcript abundance following Bge (*B*. *glabrata* embryonic cell line) exposure to parasite larval transformation products (LTP).

**Conclusions/Significance:**

The presence of a functional DNA methylation machinery in *B*. *glabrata* as well as the modulation of these gene products in response to schistosome products, suggests a vital role for DNA methylation during snail development/oviposition and parasite interactions. Further deciphering the role of this epigenetic process during *Biomphalaria*/*Schistosoma* co-evolutionary biology may reveal key factors associated with disease transmission and, moreover, enable the discovery of novel lifecycle intervention strategies.

## Author summary

Members of the genus *Biomphalaria* represent air-breathing (pulmonate) aquatic gastropod molluscs of great medical importance. The majority of species are obligatory intermediate hosts of the trematode flatworm *Schistosoma mansoni*, a pathogen responsible for the devastating neglected tropical disease schistosomiasis. Since the spread of the disease is governed by the temperature tolerance of its molluscan host, the envisaged rise in global temperatures will allow for the further expansion of the snail outside the native range into temperate regions. While schistosomiasis is currently predominantly controlled by praziquantel-mediated chemotherapy of infected individuals, novel strategies are needed in the longer term. The availability of the new *Biomphalaria glabrata* genome importantly now enables the design of next-generation schistosomiasis control strategies focused on the intermediate host. Here, using a variety of diverse approaches, we functionally characterise the critically important molecular process DNA methylation in *B*. *glabrata*, which is mediated by a suite of biological participants and is involved in a wide range of metazoan functions. Importantly, we confirm the presence of 5mC within the snail’s genome using global as well as locus-specific methodologies and we further provide the first evidence for a *S*. *mansoni-*provoked modulation of the intermediate’s host DNA methylation system.

## Introduction

With over 200 million people at risk of infection and approximately 200,000 deaths per year, schistosomiasis is the second most significant human parasitic disease on the planet [1]. This devastating and chronic illness, caused by trematode flatworms, is endemic across 78 countries of tropical and subtropical regions, with the majority of cases occurring in sub-Saharan Africa [1]. The prevalence of schistosomiasis depends on the geographical range of susceptible snail species, which serve as the obligatory intermediate host of the parasite. Three genera of pulmonate snails, *Bulinus*, *Oncomelania* and *Biomphalaria*, represent the most important intermediate hosts of medically important schistosome species (*Schistosoma haematobium*, *Schistosoma japonicum* and *Schistosoma mansoni* respectively). Anthropogenic activities, such as the construction of dams or development of irrigation schemes are commonly responsible for the population expansion of these snails [2,3] and, hence, result in the spread of this neglected tropical disease into previously unaffected regions. The high reproductive rate of these monoicous snails and their tolerance to temperature fluctuations [4] are additional factors contributing to further expansion into new geographical ranges. Indeed, *Biomphalaria spp*. have recently been found in the Ukraine [5], as well as Romania [6] and a *Bulinus sp*. has been documented in Corsica [7]. This spread northward into more temperate climates will likely accelerate based on global climate change predictions, thereby facilitating the spread of the ferrying disease [8,9].

Despite the success of intermediate host elimination in restricted schistosomiasis-endemic areas via chemical [10] or biological [11–13] measures, large-scale eradication has been difficult to implement [14]. In the absence of a prophylactic vaccine and the challenges associated with sustaining single-compound, anti-schistosomal chemotherapy [15,16], the future of integrated schistosomiasis control will increasingly rely on developing novel strategies to eliminate the intermediate host. However, in order to accomplish this objective, a deeper understanding of the intermediate host’s underlying biology and molecular processes is urgently needed [17].

In metazoans, epigenetic processes, such as those facilitated by DNA methylation, play an important and well-recognised role in basic biological phenomena including development, genome stability and phenotypic plasticity [18,19]. While our current understanding of DNA methylation has been transformed by vertebrate studies, there likely are significant differences in the conservation and function of the underlying DNA methylation machinery components in invertebrates; these are slowly being unravelled across phyla [20–23]. Within molluscs, the role of DNA methylation has only been extensively investigated in the economically important Pacific oyster *Crassostrea gigas* [24] where it was recently found that intragenic regions of moderately expressed genes and derived mobile genetic elements are predominantly targeted by this epigenetic machinery [25]. Expanding DNA methylation studies to other molluscan species would increase our understanding of this important epigenetic process within the phylum.

Here, owing to the biomedical importance of schistosomiasis and the need to further understand the molecular biology of an intermediate host responsible for disease transmission, we characterise the core DNA methylation machinery components found within the *B*. *glabrata* genome. The components identified include a maintenance DNA methyltransferase (BgDNMT1), a DNA/tRNA methyltransferase (BgDNMT2) and a methyl-CpG-binding domain protein (BgMBD2/3). Detecting DNMT and MBD activity in two different *B*. *glabrata* strains suggest that these core DNA methylation machinery components are functional, with BgDNMT1/BgDNMT2 likely responsible for the 5-methyl cytosine (5mC) modifications observed here, in addition to previous studies [26,27]. BgDNMT1 and BgMBD2/3 transcription is elevated in gonadal tissues, as well as in response to *S*. *mansoni* parasite products, indicating a role for this epigenetic process in both snail reproduction and parasite interactions. 5-azacytidine mediated inhibition of *B*. *glabrata* oviposition further supports a physiological role for DNA methylation in reproductive biology. Novel anti-schistosomal strategies targeting these DNA methylation machinery components await further investigations as an element of future integrated schistosomiasis control efforts.

## Materials and methods

### Biomphalaria glabrata

Several different *B*. *glabrata* (Bg) isolates used in this study include the NMRI (Naval Medical Research Institute) strain, the BB02 (*B**iomphalaria* from Barreiro, Brazil caught in 2002) strain, the BgBRE strain originally sampled in Recife in 1975 (Brazil), and a pigmented hybrid line obtained from Prof Michael Doenhoff’s laboratory (Nottingham University) produced by crossing numerous known susceptible isolates (Bg-Swansea, Bg-Brazil, Bg-Egypt and Bg-Belo Horizonte). Bg-Swansea snails (provenance unknown) were obtained in the early 1990s from Dr B. James of Swansea University. Bg-Belo Horizonte snails were originally collected in Belo Horizonte (1967) by W. Haas (University of Erlangen, Germany). Bg-Egypt snails (provenance unknown) were obtained from the Behring Institute for Medical Research in 1980. Bg-Brazil snails were collected in Brazil in the early 1970s and obtained from Colonel W. Radke.

### Identification of *B*. *glabrata* DNMT and MBD homologs

Full-length *B*. *glabrata* DNMT and MBD homologs were predicted by performing tBLASTn searches of the snail’s genome v4.3 using a range of DNMT (*Mus musculus* DNMT1—GenBank: P13864.5, *Apis mellifera* DNMT1—GenBank: NP_001164522.1, *Ciona intestinalis* DNMT1—XP_002122948.1) and MBD (*Aplysia californica*—GenBank: XP_005103642.1, *Crassostrea gigas* MBD2/3—GenBank: EKC32831.1) query sequences. The exon-intron structures of *Lottia gigantea* DNMT1 (transcript name: 114987), DNMT2 (transcript name: 119453) and MBD2/3 (transcript name: 112523; all [28]) were used to finalise the *B*. *glabrata* gene structures.

### *B*. *glabrata* DNA methylation machinery cloning

Two day-fasted, laboratory bred specimens of NMRI strain were dissected and RNA subsequently isolated using TRIzol Reagent (Invitrogen) according to the manufacturer’s protocol. Following treatment with DNaseI (Ambion), 1 μg of RNA was reverse-transcribed using random hexamer primers and SuperscriptIII (Invitrogen). Oligonucleotide pairs (S1 Table), designed from the predicted sequences, were used to amplify full-length (ATG to stop) BgMBD2/3 (729 bp) and BgDNMT2 (1182 bp) sequences from cDNA derived from the head/foot of an individual NMRI snail. PCR products were subsequently cloned into pGEM-T Easy vector (Promega) before being sequenced. In the case of BgDNMT1, a 1652 bp product (containing the catalytic domain within its C-terminus) was amplified and subjected to pGEM-T Easy vector cloning as well as DNA sequencing. Following sequence confirmation, the translated sequences of BgDNMT1, BgDNMT2 and BgMBD2/3 were subsequently submitted to a Pfam domain search [29] and the identified domains of BgDNMT1 (PF12047, PF02008, PF01426, PF00145), BgDNMT2 (PF00145) and BgMBD2/3 (PF01429, PF14048) were extracted. Furthermore, the presence of a nuclear localisation signal (NLS) within the ORF of BgDNMT1 was examined and confirmed using cNLS mapper [30].

### Sequence alignments and phylogenetic analyses

Multiple sequence alignments of BgDNMT1, BgDNMT2 and BgMBD2/3 were generated using MUSCLE v3.8 [31]. The catalytic domain (PF00145) of BgDNMT1 and BgDNMT2 was aligned to the sequences of the following organisms (GenBank accession number): *A*. *californica* DNMT2 (XP_005095276.1), *L*. *gigantea* DNMT2 (transcript name: 119453 [28]), *Capitella teleta* DNMT2—ELU13416.1, *Helobdella robusta* DNMT2 (transcript name: 89038 [28], *S*. *mansoni* DNMT2 (HM991456.1), *A*. *mellifera* DNMT2 (XP_393911.3), *M*. *musculus* DNMT2 (AAC53529), *A*. *californica* DNMT1 (XP_005104649.1 [28]), *L*. *gigantea* DNMT1 [28], *C*. *teleta* DNMT1a (ELT93682.1), *H*. *robusta* DNMT1 (transcript name: 116156 [28]), *A*. *mellifera* DNMT1 (NP_001164522.1) and *M*. *musculus* DNMT1 (P13864.5). In the case of BgMBD2/3, an alignment was created using the following sequences (GenBank accession number): *A*. *californica* (XM_005103585.1), *L*. *gigantea* MBD2/3 (transcript name: 112523 [28]), *C*. *gigas* MBD2/3 (EKC32831.1), *H*. *robusta* MBD2/3 (transcript name: 185546 [28]), *C*. *teleta* MBD2/3 (ELT95247.1), *S*. *mansoni* (HM991455), *Paragonimus westermani* MBD2/3 [32], *S*. *japonicum* MBD2/3 (AAW26585.1), *Hymenolepis microstoma* MBD2/3 [32], *Echinococcus multilocularis* MBD2/3 [32], *Echinococcus granulosus* DNMT2 [32], *Taenia solium* MBD2/3 [32], *Schmidtea mediterranea* MBD2/3 [32], *Hemicentrotus pulcherrimus* MBD2/3 (EU590662), *M*. *musculus* MBD2 (NP_034903), *M*. *musculus* MBD3 (NM_013595).

For phylogenetic analysis of BgMBD2/3, BgDNMT1 and BgDNMT2 based on Bayesian (MrBayes v3.1.2 [33]) and Maximum Likelihood (MEGA v5.2.2 [34]) approaches, amino acid sequences were aligned using MUSCLE v3.8 [31]. The six highly conserved motifs within the catalytic domain (PF00145) of BgDNMT2 and BgDNMT1 were aligned with sequences from (GenBank accession number): *A*. *californica* DNMT2 (XP_005095276.1), *L*. *gigantea* DNMT2 (transcript name: 119453 [28]), *C*. *teleta* DNMT2 (ELU13416.1), *H*. *robusta* DNMT2 (transcript name: 89038 [28]), *C*. *intestinalis* DNMT2 (XP_002128135.1), *M*. *musculus* DNMT2 *(*AAC53529.1), *S*. *mediterranea* DNMT2 [32], *E*. *multilocularis* DNMT2 [32], *S*. *mansoni* DNMT2 (HM991456.1), *Fasciola hepatica* DNMT2 [32], *A*. *mellifera* DNMT2 (XP_393911.3), *Culex quinquefasciatus* DNMT2 (XP_001867327.1), *C*. *intestinalis* DNMT3a (XP_002123461.1), *M*. *musculus* DNMT3a (O88508.2) *M*. *musculus* DNMT3b (O88509.2), *H*. *robusta* Dnmt3 (transcript name: 162653 [28]), *A*. *mellifera* DNMT3 (NP_001177350.1), *L*. *gigantea* Dnmt3 (transcript name: 171288 [28]), *Bombyx mori* DNMT1 (NP_001036980.1), *A*. *mellifera* DNMT1 (NP_001164522.1), *H*. *robusta* DNMT1 (transcript name: 116156 [28]), *A*. *californica* DNMT1 (XP_005104649.1 [28]), *C*. *teleta* DNMT1a (ELT93682.1), *L*. *gigantea* DNMT1 [28], *C*. *intestinalis* DNMT1 (XP_002122948.1) and *M*. *musculus* DNMT1 (P13864.5). For the phylogenetic analysis of BgMBD2/3, an amino acid sequence alignment of the following MBD sequences was used (GenBank accession number): *Clonorchis sinensis* MBD2/3 [32], *Opisthorchis viverrini* MBD2/3 [32], *S*. *mansoni* (HM991455), *P*. *westermani* MBD2/3 [32], *S*. *japonicum* MBD2/3 (AAW26585.1), *F*. *hepatica* MBD2/3 [32], *E*. *multilocularis* MBD2/3 [32], *E*. *granulosus* DNMT2 [32], *Taenia solium* MBD2/3 [32], *H*. *microstoma* MBD2/3 [32], *S*. *mediterranea* MBD2/3 [32], *Macrostomum lignano* MBD2/3 [32], *H*. *pulcherrimus* MBD2/3 (EU590662), *A*. *californica* (XM_005103585.1), *L*. *gigantea* MBD2/3 (transcript name: 112523 [28]), *C*. *gigas* MBD2/3 (EKC32831.1), *C*. *teleta* MBD2/3 (ELT95247.1), *Xenopus laevis* MBD3 (BAC22082.1), *M*. *musculus* MBD3 (NM_013595), *X*. *laevis* MBD2 (NP_001083787.1), *M*. *musculus* MBD2 (NM_010773), *X*. *laevis* MBD1 (NP_001104183.1), *M*. *musculus* MBD1 (NM_013594), *X*. *laevis* MeCP2 *(*AAD03736.1), *M*. *musculus* MeCP2 (NM_010788), *Xenopus tropicalis* MBD4 (NP_001037916) and *M*. *musculus* MBD4 (NM_010774). In the case of the MBD homologs, ambiguously aligned regions were removed with Gblocks v0.91b [35]. Maximum Likelihood analysis was conducted with the Jones-Taylor-Thornton (JTT) substitution model and 500 bootstrap replicates. Bayesian inferences were computed using the WAG substitution model, performing four independent Markov Chain Monte Carlo runs for 1,000,000 generations. Graphical output of the final Bayesian consensus phylograms was then obtained via Figtree v1.3.1 [36] and further manual annotations were made in Adobe Illustrator v13.0.2.

### RNA-Seq: RNA isolation, library preparation and sequencing

First, a diverse set of tissues including albumen gland (AG), buccal mass (BUC), central nervous system (CNS), digestive gland/hepatopancreas (DG/HP), head/foot (FOOT), heart/amebocyte producing organ (HAPO), kidney (KID), mantle edge (MAN), ovotestes (OVO), salivary glands (SAL), stomach (STO) and terminal genitalia (TRG) was dissected from adults of the *B*. *glabrata* BB02 strain and pooled from 4–5 individual snails. Thereafter, total RNA was isolated using TRIzol Reagent (Invitrogen) and subsequently DNase treated following the manufacturer’s protocol (Ambion). Poly(A)+ RNA was isolated from total RNA (Ambion MicroPoly(A)Purist kit), quality controlled using an Agilent 2100 Bioanalyzer (RIN score = 7–8) and used to generate a non-normalised cDNA library by the NuGEN Ovation RNA-Seq System V2 (NuGEN). Finally, each cDNA library was sequenced on an Illumina HiSeq instrument (~36Gb per lane). The raw RNA-Seq reads of each sample are available in the NCBI BioProject repository (PRJNA12879).

### RNA-Seq: Quality control and differential expression analysis

Prior to mapping of the raw sequence data, adaptor and primer sequences were removed from the Illumina paired-end reads with FASTX-Clipper [37] and a quality control check was performed using FastQC [38]. Thereafter, reads were mapped to the *B*. *glabrata* genomic scaffolds available at VectorBase [39] with TopHat2 [40]. Subsequently, the Samtools mpileup program [41] was employed for SNP/INDEL calling and the variants encountered were filtered for quality as previously described in Jia *et al*. [42]. A normalised gene expression count matrix was generated using the R statistical programming language v3.1.2 [43], the Bioconductor packages GenomicRanges and GenomicAlignments [44], as well as DESeq2 following the protocol of Anders and colleagues [45]. DESeq2 was also used to conduct differential expression analyses (cut-offs included a 10% false discovery rate [46] and a minimum log_2_ fold change of 1 amongst different snail tissue types [45]).

### RNA-Seq: Gene annotation and association network

Using BLAST2GO [47], gene ontology (GO) terms [48] were assigned to differentially expressed transcripts and the relationships between genes was represented as a network where a node (vertex) represents a gene and a line (edge) connecting two genes represents neighbours [49]. Using the igraph library [50] in R [51], differentially expressed genes were represented in the form of a graph. Two genes are associated (i.e. were connected by a line) if they shared a ‘Biological Process’ GO term category and their expression profiles were correlated (0.6 ≤ Pearson Correlation ≤ -0.6).

### qRT-PCR confirmation of RNA-Seq data

Samples from AG, STO, FOOT, DG/HP and OVO were dissected from 3–4 BgBRE snails under a binocular dissection microscope (three biological replicates for each tissue). Haemocytes from 10 snails were collected from haemolymph after centrifugation at 10,000 x g for 10 min at 4°C. Total RNA was subsequently isolated from the five different tissues and haemocytes using TRIzol Reagent (Invitrogen) according to the manufacturer’s protocol. Thereafter, RNA samples (10 μg) were treated with DNaseI (Ambion) and 1 μg was reverse-transcribed using random hexamer primers and Revertaid H minus M-MuLV reverse transcriptase (Fermentas). qRT-PCR was then performed on cDNAs (diluted 20-fold with nuclease-free water) using the Light Cycler System 480 (Roche). Primer sequences used for amplification of *Bgmbd2/3* (BgMBD2/3 qRT-PCR1), ribosomal protein *BgS19* and *Bgdnmt1* can be found in S1 Table. Ct-values of the target genes were normalised to the transcript level of the reference gene *BgS19* (GenBank: CK988928) using the Pfaffl method as described in Chalmers *et al*. [52]. Each qRT-PCR experiment was performed at least twice and biological duplicates were used for each tissue and technical triplicates performed for every qRT-PCR reaction. In the case of haemocytes, technical duplicates of one sample were used.

### 5-azacytidine (5-AzaC) treatment of *B*. *glabrata* snails

NMRI *B*. *glabrata* snails (1–1.2 mm in size) were maintained in artificial freshwater (0.378 mM CaCl_2_, 0.5 mM MgSO_4_-7H_2_O, 0.025 mM K_2_SO_4_, 0.5 mM NaHCO_3_, 0.0002 mM FeCl_3_-6H_2_O in dI water) in the presence (491μM) or absence of the demethylating agent 5-AzaC (Sigma) at 28°C for eight days. Two replicate experiments were performed (experiment one = 10 snails/condition; experiment two = 12 snails/condition) with the 5-AzaC replaced at day four and the total number of egg sacs laid/condition recorded at day eight. The Student’s two-tailed *t*-test was used to determine statistical differences in egg sacs laid between the treatments.

### BgDNMT and BgMBD2/3 enzymatic assays

Nuclear proteins were extracted from the head/foot of starved NMRI and pigmented hybrid adult snails (20 mg of tissue derived from 4 individuals/strain) using the Epiquik Nuclear Extraction Kit (Epigentek). DNA methyltransferase activity contained within 7 μg of nuclear protein extracts was subsequently measured using the EpiQuik DNA Methyltransferase Activity/Inhibition Assay Kit (Epigentek). Fluorescent readings (530_EX_/590_EM_ nm) were obtained using a POLARstar Omega (BMG Labtech) microtiter plate reader and data were normalised as previously described [27]. Snail MBD activity was measured in 10 μg of nuclear protein extracts using an EpiQuik MBD2 Binding Activity/Inhibition Assay Kit (Epigentek). Fluorescent readings were obtained as above and data was subsequently normalised to both negative control (10 μg of BSA) and positive control (MBD2, supplied by kit) samples. Data are presented as means ± standard deviation (SD) and each assay was repeated at least twice.

### ELISA-based detection of 5mC

gDNA was isolated from a pool of either four starved individual NMRI or pigmented hybrid snails using the DNeasy Blood and Tissue Kit (Qiagen). A treatment step with RNase (Invitrogen) followed and 5mC abundance was subsequently fluorometrically determined from 100 ng of RNA-free gDNA using the SuperSense methylated DNA Quantification Kit (Epigentek) as previously described [27]. The assay was performed in duplicate, repeated twice and readings are presented as means ± standard deviation (SD). 5mC abundance was calculated based on the *B*. *glabrata* genome GC content (35%) using the following equation:

$$5mC\;percentage=\frac{\frac{\left(sample-neg\;control\right)}{GC\;content}}{RFU(pos\;control-neg\;control)\times 10}\times 100\%$$

### BS-PCR: Locus-specific detection of 5mC

Bisulfite conversion was performed as previously described by Fneich *et al*. [26]. Briefly, 300 ng gDNA (derived from a pool of 10 individual snails of the BgBRE strain) was denatured with 3M NaOH and subsequently treated with a solution of sodium-bisulfite and hydroquinone at pH 5 in the dark for 4 hr at 55°C. Thereafter, the gDNA was desalted (Amicon Ultra column, UFC510024 Millipore), desulfonated by the addition of 350 μl of 0.1M NaOH and finally dissolved in 50 μl of 10 mM Tris/Cl (pH 8). A nested PCR was then performed to amplify regions of the *Bg14-3-3* (Scaffold 8484:17058–17923) gene. Primer pairs were designed using MethPrimer [53] on genomic sequences extracted from the preliminary genome assembly v4.3 [54] as indicated in S1 Table. The initial PCR amplification was performed using 1 μl of the bisulfite converted gDNA samples as templates with external primer set as follows: 94°C for 2 min, 5 cycles of 94°C for 1 min, 46°C for 2 min and 72°C for 3 min, followed by 25 cycles of 94°C for 30 sec, 46°C for 2 min and 72°C for 1:30 min and finally 72°C for 10 min. The nested PCR was performed on a 10 fold dilution of the first PCR product using the internal primer set in the same condition as for the first PCR except for the annealing temperature which was increased to 50°C. The subsequent PCR reaction was performed in 25 μl using 1.25 units of Go Taq DNA polymerase (Promega), dNTPs at 0.4 μM for each deoxynucleotide and primers at 0.4 μM. PCR products were separated by electrophoresis through 1% agarose gels to check for the specific amplification of each target gene. For high-resolution analysis, 1 μl of each PCR product was cloned into pCR4 (TOPO TA Cloning kit, Invitrogen) and positive clones were sequenced with vector specific primers (S1 Table) using GenoScreen sequencing facilities (Campus de l'Institut Pasteur de Lille, France). Sequences obtained from the bisulfite treated gDNA were aligned with their respective genomic reference sequence in Bioedit v7.2.5 [55] to identify methylated cytosines. MethTools v2.0 software [56] was used to generate a graphical view of each target region containing the methylated sites. The Whole Genome Bisulfite-Seq (WGBS) data set, performed as part of the *B*. *glabrata* genome project (Genome Publication, under review), was then inspected for the presence of methylated CpG sites within the *Bg14-3-3* gene using the genome browser IGV v2.3 [57].

### qRT-PCR of control vs. LTP-treated Bge cells

In order to test the effects of naturally produced larval products on expression of the epigenome machinery in snail cells, the *B*. *glabrata* embryonic (Bge) cell line was exposed *in vitro* to *S*. *mansoni* larval transformation products (LTP; [58]) for 24 hr at 26°C and subjected to qRT-PCR analyses. Briefly, mRNA was isolated from control and LTP-treated Bge cells as well as Bge cells treated with *S*. *mansoni* larval transformation products (LTP) as previously described [59]. qRT-PCR was subsequently employed to investigate *Bgdnmt1* and *Bgmbd2/3* transcript abundance between samples derived from LTP-treated versus control cells. Amplifications were performed on a StepOnePlus (ABI) qRT-PCR machine using SYBR Green (ABI) chemistry; primer sequences can be found in S1 Table (BgMBD2/3 qRT-PCR1). The Ct-values of the target genes were normalised to the transcript level of the reference gene Actin (GenBank: Z72387; [58]) using the Pfaffl method as described in Chalmers *et al*. [52]. Results are based on two biological replicates and each qRT-PCR reaction was performed in technical duplicates. No amplification was observed in negative control reactions (H_2_O instead of cDNA template).

## Results and discussion

### Sequence confirmation and characterisation of BgMBD2/3

**Fig 1:**
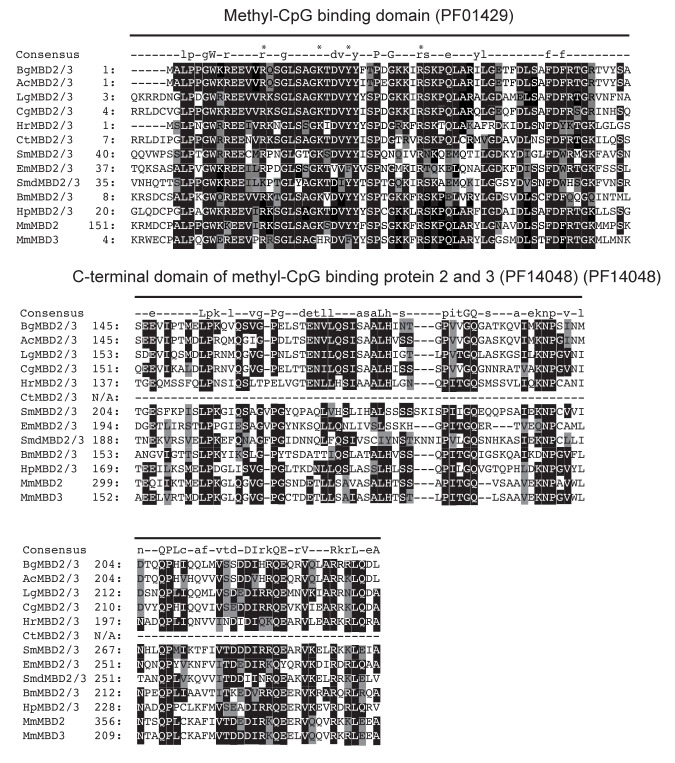
*B*. *glabrata* contains a methyl-CpG-binding protein, BgMBD2/3, which shares high sequence similarity with eukaryotic MBD proteins. Alignment of methyl-CpG binding domain (PF01429) and C-terminal domain of methyl-CpG-binding domain protein 2 & 3 (PF14048) regions using MUSCLE [31]. Abbreviations Bg, Ac, Lg, Cg, Hr, Ct, Sm, Em, Smed, Bm, Hp, and Mm refer to *B*. *glabrata*, *A*. *californica*, *L*. *gigantea*, *C*. *gigas*, *H*. *robusta*, *C*. *teleta*, *S*. *mansoni*, *E*. *multilocularis*, *S*. *mediterranea*, *B*. *mori*, *H*. *pulcherrimus* and *M*. *musculus*. An asterisk in the upper line indicates functionally important amino acids within the methyl binding domain region. Numbers at the beginning of each line represent amino acid positions and at each position the most conserved residues are further shaded in black, semi-conserved residues are highlighted grey and non-conserved amino acids are kept white. 'Consensus' represents the Pfam consensus sequence of each domain where conserved amino acids (50–79%) are indicated by lower-case and highly conserved residues (> 80%) by upper-case letters. Missing amino acid residues, not present in the truncated CtMBD2/3 candidates, are indicated by a ‘N/A’.

A tBLASTn search against the preliminary *B*. *glabrata* genome assembly v4.3 [54], using known molluscan MBD homologs (*A*. *californica*—GenBank: XP_005103642.1, *C*. *gigas* MBD2/3—GenBank: EKC32831.1), revealed the presence of a single MBD protein. A subsequent BLASTp search against the NCBI database using the predicted sequence demonstrated 75% identity with the *A*. *californica* homolog (NP_00510364.2; E-value of 2e^-156^). This confirms findings by Fneich *et al*. [26], who had previously identified a partial MBD2/3 homolog in the preliminary *B*. *glabrata* genome assembly, as well as in available RNA-Seq datasets. The transcript sequence encoding the 242 aa predicted ORF of the *B*. *glabrata* MBD2/3 (BgMBD2/3) homolog was subsequently amplified from adult NMRI head/foot cDNA and PFAM domain search analysis of the cloned product (GenBank: KJ951055) revealed the presence of a N-terminal MBD domain (PF01429), as well as a C-terminal domain conserved amongst proteins of the MBD2 and MBD3 family (PF14048). Multiple sequence alignment of BgMBD2/3 with related proteins further demonstrated high levels of sequence conservation over the entire N-terminal MBD domain and high similarity to invertebrate-specific MBD2/3 proteins, as well as the murine MBD2 and MBD3 homologs (Fig 1).

**Fig 2:**
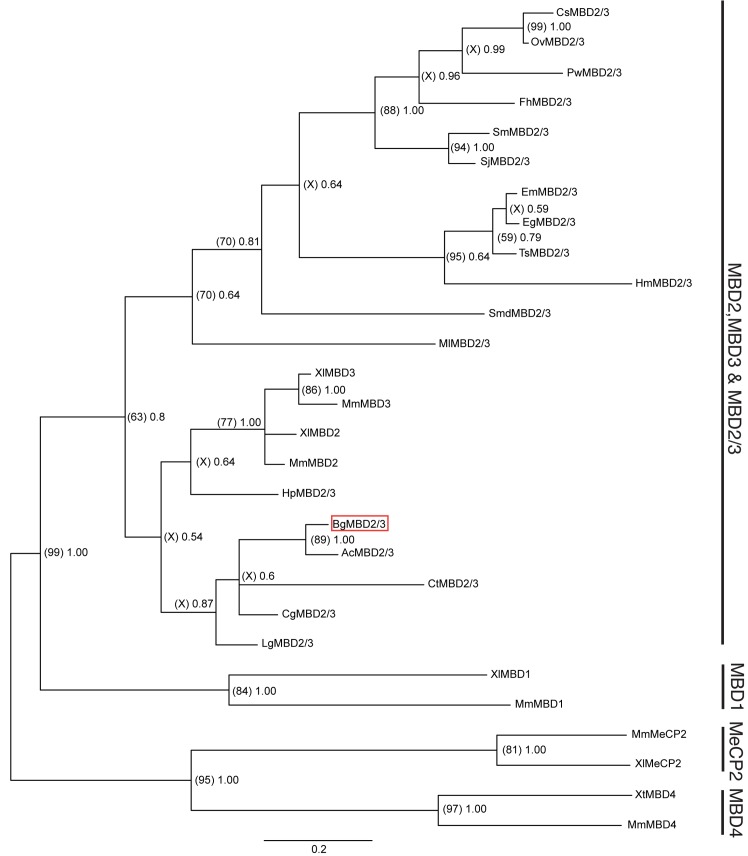
BgMBD2/3 represents a homolog of the invertebrate-specific MBD2/3 clade. Phylogenetic reconstruction based on Bayesian (Mr Bayes v3.1.2) and Maximum Likelihood (MEGA v5.2.2) methods were estimated from a multiple sequence alignment based on the MBD domain of 28 eukaryotic MBD sequences. Notations Cs, Ov, Pw, Sm, Sj, Fh, Em, Eg, Ts, Hm, Smed, Hr, Lg, Ac, Cg, Ct, Hp, Mm, Xl and Xt refer to *C*. *sinensis*, *O*. *viverrini*, *P*. *westermani*, *S*. *mansoni*, *S*. *japonicum*, *F*. *hepatica*, *C*. *sinensis*, *E*. *multilocularis*, *E*. *granulosus*, *T*. *solium*, *H*. *microstomum*, *S*. *mediterranea*, *M*. *lignano*, *H*. *robusta*, *L*. *gigantea*, *A*. *californica*, *C*. *gigas*, *C*. *telata*, *H*. *pulcherrimus*, *M*. *musculus*, *X*. *laevis* and *X*. *tropicalis*. A graphical output of the Bayesian consensus phylogram was obtained via Figtree v1.3.1 [36] and BgMBD2/3 is included within a red box. Node labels within parentheses represent percentage bootstrap support values from Maximum Likelihood analysis (500 bootstrap replicates performed using the JTT model), while those outside parentheses represent Bayesian posterior probability support values (based on performing four independent Markov Chain Monte Carlo runs for 1,000,000 generations using the WAG model). Only nodes with Bayesian posterior probability support values > 0.5 are shown.

Furthermore, unlike the mammalian MBD3, which contains limited 5mC binding capability due to a single amino acid substitution [60], the presence of crucial residues (indicated in alignment by asterisk: R14, K22, Y26, R36), essential for the binding of the protein to methylated DNA [32], enables us to propose that the snail homolog would be a functional member of this protein family. The presence of a C-terminal region unique to MBD2 and MBD3 proteins (PF14048), in addition to the absence of a glycosylase domain (characteristic for MBD4) and Zn-finger motif (found in MBD1) suggests that the *B*. *glabrata* MBD is a novel MBD2/3 homolog. Phylogenetic analyses based on Bayesian and Maximum Likelihood inferences of BgMBD2/3 with characterised MBDs, provides additional supporting evidence that the *B*. *glabrata* MBD is a *de facto* MBD2/3 homolog (Fig 2).

As expected for an invertebrate organism, BgMBD2/3 clusters with invertebrate-specific MBD2/3 proteins as well as closely related vertebrate MBD2 and MBD3 members. Nevertheless, the *B*. *glabrata* MBD2/3 homolog is placed with great reliability (bootstrap value of 99, posterior probability of 1.00) outside a distinct clade of vertebrate MeCP2, MBD1 and MBD4 homologs and is most similar (bootstrap value of 89, posterior probability of 1.00) to another molluscan MBD2/3 exemplar (*A*. *californica* AcMBD2/3).

### Sequence confirmation and bioinformatics characterisation of BgDNMT2 and BgDNMT1

**Fig 3:**
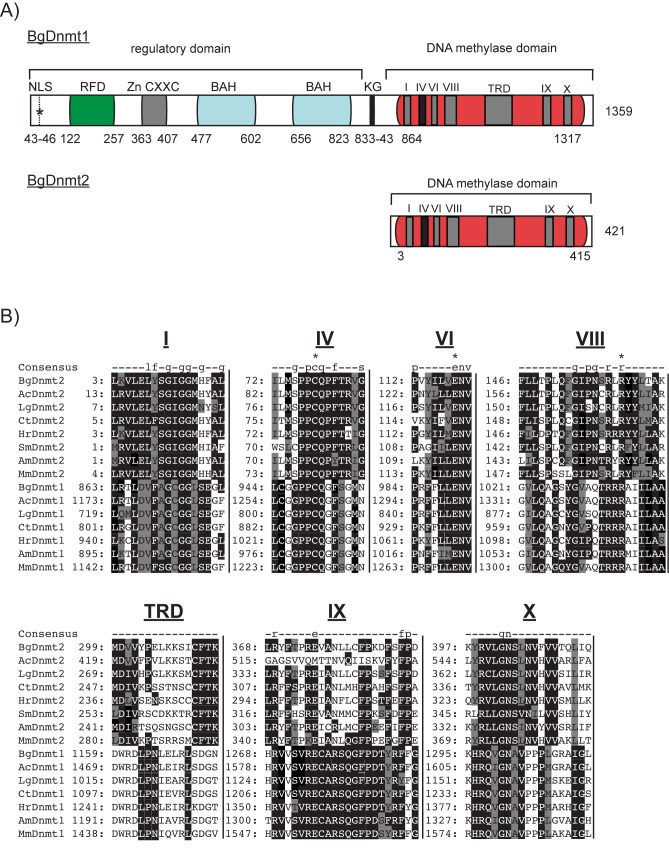
The *B*. *glabrata* genome encodes both DNA methyltransferase 1 (BgDNMT1) and 2 (BgDNMT2) family members. **A) Cartoon representation of BgDNMT1 and BgDNMT2.** C-terminal catalytic DNA methylase domains (PF00145) present in BgDNMT1 and BgDNMT2 are highlighted in red. Roman numerals (I, IV, VI, VIII, IX & X) within the DNMT domain denote the highly conserved motifs and TRD represents the target recognition domain. In BgDNMT1, this DNA methylase domain is linked to a N-terminal regulatory region via a KG linker (black vertical bar; amino acid position 833–843). The regulatory domain contains a nuclear localisation signal (NLS), indicated with an asterisk, a cytosine-specific DNA methyltransferase replication foci domain (RFD; PF12047), a Zinc Finger CXXC Domain (PF02008) and two bromo-adjacent homology (BAH) domains (PF01426), coloured in green, grey and turquoise respectively. Numbers represent amino acid positions. **B) *B*. *glabrata* DNMT homologs share extensive sequence similarity throughout the DNA methylase catalytic domain (PF00145).** A multiple sequence alignment of the invariant C-termini (as predicted by Pfam domain search) of BgDNMT1 and BgDNMT2 with homologous enzymes was performed using MUSCLE (Multiple Sequence Comparison by Log-Expectation; [36]). The six highly conserved motifs of the catalytic domain are indicated with Roman numerals (I, IV, VI, VIII, IX & X) and TRD marks the Target Recognition Domain. Functionally important residues are highlighted with an asterisk and numbers at the beginning of each motif represent the amino acid positions. The most conserved residues are further shaded in black, semi-conserved residues are highlighted grey and non-conserved amino acids are white. 'Consensus' represents the Pfam consensus sequence of each domain where conserved amino acids (50–79%) are indicated by lower-case, and highly conserved residues (> 80%) by upper-case letters.

Following the identification of a MBD homolog within the *B*. *glabrata* genome (BgMBD2/3), a subsequent tBLASTn against the genome assembly using eukaryotic Dnmt homologs (*M*. *musculus* DNMT1—GenBank: P13864.5, *A*. *mellifera* DNMT1—GenBank: NP_001164522.1, *C*. *intestinalis* DNMT1—XP_002122948.1) revealed the presence of a Dnmt1 as well as a Dnmt2 candidate in *B*. *glabrata* (Fig 3).

Thereafter, a BLASTp search against the NCBI database with the predicted *B*. *glabrata* DNA methyltransferase sequences revealed 54% identity of BgDNMT2 with the *L*. *gigantea* homolog (XP_009052047.1; 2e^-134^) and 75% identity of BgDNMT1 with the *A*. *californica* DNMT1 sequence (XP_00509576.1; E-value 0.0). Using the preliminary genome assembly and available RNA-Seq datasets, partial DNMT1 and DNMT2 sequences had previously been identified by Fneich *et al*. (2013). Similar to BgMBD2/3, the sequences of the two predicted DNA methyltransferases were confirmed using cDNA derived from the head/foot of adult NMRI snails. We were able to confirm the complete 393 aa ORF of BgDNMT2 (GenBank: KJ951056), as well as a 550 aa C-terminal region of BgDNMT1, which includes the catalytic domain. A subsequent Pfam domain search revealed the presence of a DNA methylase domain (PF00145) containing six highly conserved motifs (I, IV, VI, VIII, IX and X) and the target recognition domain (TRD) in both BgDNMT2 (aa residues 3–415) and BgDNMT1 (aa residues 863–1,314) members (Fig 3A). In contrast, a regulatory domain containing a nuclear localisation signal (NLS), a cytosine-specific DNA methyltransferase replication foci domain (RFD; PF12047), a Zinc Finger CXXC domain (PF02008) and two bromo-adjacent homology (BAH) domains (PF01426) were only found in BgDNMT1 (Fig 3A).

**Fig 4:**
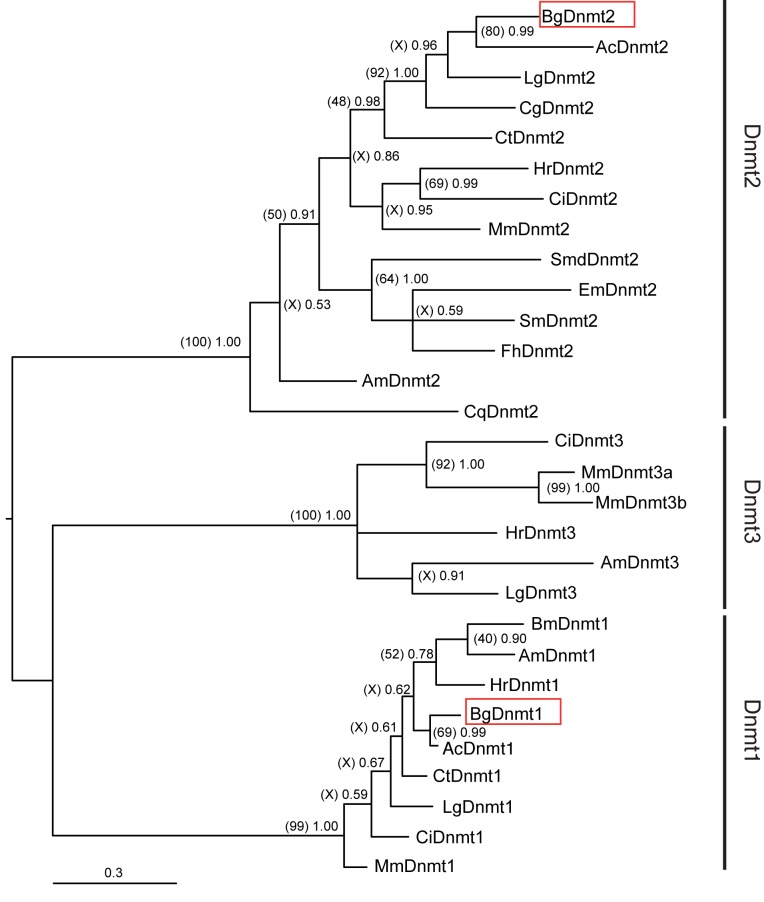
*B*. *glabrata* DNMT homologs are novel members of the DNMT enzyme family. Phylogenetic relationships based on Bayesian (Mr Bayes v3.1.2) and Maximum Likelihood (MEGA v5.2.2) approaches, were inferred from a multiple sequence alignment of the six highly conserved motifs within the catalytic domain (PF00145) from 29 taxa using MUSCLE [31]. BgDNMT1 and BgDNMT2 are indicated by red boxes. Abbreviations Bg, Ac, Lg, Ct, Hr, Ci, Mm, Smd, Em, Sm, Fh, Am and Cq relate to *B*. *glabrata*, *A*. *californica*, *L*. *gigantea*, *C*. *gigas*, *C*. *teleta*, *H*. *robusta*, *C*. *intestinalis*, *M*. *musculus*, *S*. *mediterranea*, *E*. *multilocularis*, *S*. *mansoni*, *F*. *hepatica*, *A*. *mellifera* and *C*. *quinquefasciatus*. The Bayesian analysis consensus tree is illustrated (Figtree v1.3.1, [36]) with branch lengths signifying distance between taxa. Node labels within parentheses represent percentage bootstrap support values from Maximum Likelihood analysis (500 bootstrap replicates performed using the JTT model), while those outside parentheses represent Bayesian posterior probability support values (based on performing four independent Markov Chain Monte Carlo runs for 1,000,000 generations using the WAG model).

Subsequent alignment of BgDNMT1 and BgDNMT2 C-terminal DNA methylase domains (PF00145) with known DNMT enzymes revealed strong sequence similarity across the six most conserved motifs (I, IV, VI, VIII, IX and X) (Fig 3B). Specifically, the catalytically crucial proline/cysteine dipeptide [61] is present in both BgDNMT2 (P77 & C78) and BgDNMT1 (P949 & C950). To discriminate the two enzyme families, DNMT2-specific residue substitutions within BgDNMT2 were noted: tyrosine (Y) to phenylalanine (FXGXG) in motif I and asparagine (N) to glutamine (QXGXG) in motif VIII [61]. Moreover, the DNMT2-specific cysteine/phenylalanine/threonine (CFT) tripeptide within the target recognition domain (TRD) is uniquely present in BgDNMT2, but not in BgDNMT1. A phylogram based on sequence alignment of 29 representative eukaryotic members of all three DNA Mtase families (DNMT1, DNMT2 and DNMT3) clearly separates BgDNMT2 and BgDNMT1 into their distinct clades (Fig 4).

Despite being the most conserved of all DNA methyltransferases, the biological function of DNMT2 enzymes is highly debatable and its ability to methylate a DNA target has been questioned on numerous occasions [62,63]. Nevertheless, its dual biological activity and substrate specificity is now becoming more commonly accepted. For example, in mammals, DNMT2 predominantly serves as a tRNA methyltransferase [64]. However, in lower eukaryotes, DNMT2 commonly functions as the sole DNA methyltransferase [27,65,66]. Nevertheless, and in line with other molluscs (i.e. the pacific oyster [67]), the *B*. *glabrata* genome encodes, in addition to a DNMT2 protein, a DNMT1 homolog. The latter is commonly referred to as a maintenance DNA methyltransferase, as members of this enzyme family preferentially methylate hemimethylated DNA [68]. Unlike DNMT2 homologs, DNMT1 enzymes additionally have a large regulatory N-terminal domain comprised of several notable elements (Fig 3A). As BgDNMT1 contains these domains in the conserved order: 1) a DNMT1-replication foci domain (RFD; PF12047), a zinc finger domain (CXXC; PF02008) and two bromo adjacent homology domains (BAH; PF01426), 2) has a predicted nuclear localisation signal (NLS) between residues 40–48 (QGSAKRIKLQ) and 3) includes the KG-repeat linker ((KG)_4_; [69]) connecting the catalytic domain and N-terminal regions (between residues 833–843), we propose that this *B*. *glabrata* homolog is a functional member of this DNA methyltransferase family. Despite exhaustive searches, no DNMT3A or B homolog was found within the genome of *S*. *mansoni’*s intermediate snail host, suggesting that BgDNMT1 (and to a lesser extent BgDNMT2) functions as the main cytosine methyltransferase within this invertebrate species.

Our identification of both DNMT1 and DNMT2 (but not DNMT3) DNA methyltransferase in the *B*. *glabrata* genome is in line with results recently obtained for *A*. *californica*, but is in contrast to the detection of a full set of DNMTs (DNMT1, DNMT2 and DNMT3) in *C*. *gigas* and *L*. *gigantea* [70]. This differential inclusion/exclusion of DNMTs in molluscan genomes has also been observed in the phylum Arthropoda where some members contain all three DNA methyltransferase families (e.g. *Apis mellifera* [71] and *Nasoni spp*. [72]), others (e.g. *Locusta migratoria* [73]), *B*. *mori* [71]), *Tribolium castaneum* [74] and *Schistocerca gregaria* [75] only contain DNMT2 and DNMT1 homologs, while others (*Drosophila melanogaster* [76]) only contain a single DNMT2 enzyme responsible for all 5mC modifications. Similar to arthropods, the significance of DNMT3 exclusion in specific molluscan species (e.g. *B*. *glabrata*) awaits further investigations.

### Tissue-specific expression of *B*. *glabrata* DNA methylation machinery

**Fig 5:**
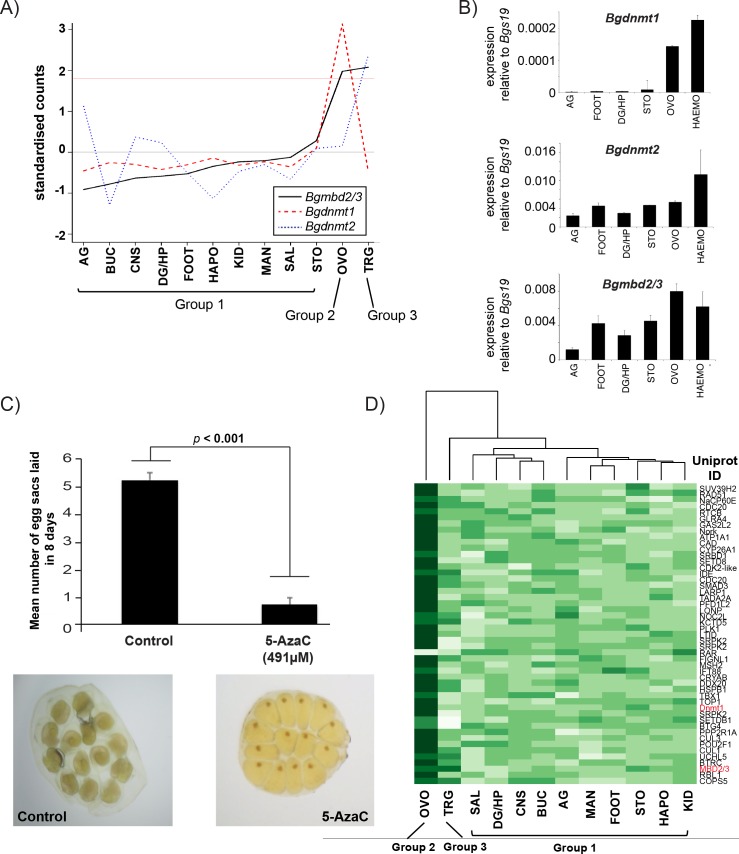
The *B*. *glabrata* DNA methylation machinery is abundantly expressed in sex tissues and haemocytes. **A) RNA-Seq analysis of the *B*. *glabrata* DNA methylation machinery in twelve snail tissues.** The normalised sequencing counts [51] for each gene of interest (i.e. *Bgmbd2/3*, *Bgdnmt1* and *Bgdnmt2*) across the twelve tissues were used to estimate sample parameters for that gene i.e. the mean and standard deviation. The twelve observations for each gene were scaled to a standardised t-distribution. These standardised counts for the three genes were plotted (y-axis) against the twelve tissues (x-axis)—the continuous the red line on the y-axis at 1.79 represents *p* < 0.05 on a t-distribution with 11 degrees of freedom. The samples were divided into three groups, i.e Group 2: ovotestes (OVO), Group 3: terminal genitalia (TRG) and Group 1: salivary glands (SAL), digestive gland/hepatopancreas (DG/HP), central nervous system (CNS), buccal mass (BUC), albumin gland **(**AG), mantle edge (MAN), head/foot (FOOT), stomach (STO), heart/APO (HAPO) and kidney (KID). Differential expression analysis, using DESeq2 [51], indicates the Group 1 vs. Group 2 and Group 1 vs. Group 3 comparisons of *Bgmbd2*/*3*, *Bgdnmt1* and *Bgdnmt2* abundance (i.e. tissue samples with data above the red line) are statistically significant for that gene of interest. **B) qRT-PCR data confirms the tissue-enriched expression of the *B*. *glabrata* DNA methylation machinery.** qRT-PCR was employed to verify the transcript abundance of *Bgdnmt1*, *Bgdnmt2* and *Bgmbd2/3* across five tissues previously analysed by RNAseq. In addition to albumin gland **(**AG), head/foot (FOOT), stomach (STO), ovotestes (OVO) and digestive gland/hepatopancreas (DG/HP), transcript abundance was also determined in haemocytes (HAEMO). Error bars represent standard deviation of the mean (SD). The Ct values of target genes were normalised to the reference gene S19 [77]. Biological duplicates were used for each tissue and technical triplicates performed for every qRT-PCR reaction. For haemocytes, only one biological sample was available. **C) 5-AzaC treatment inhibits *B*. *glabrata* oviposition.** Adult NMRI snails (10–12 individuals/condition) were incubated in the presence or absence of 491μM 5-AzaC for a total of eight days. The bar chart represents mean eggs laid/condition at day eight + standard deviation (SD). The Student’s two-tailed *t* test was performed to identify significant differences between the treatments. Images are representative of egg sacs obtained from control and 5-AzaC conditions and were taken 7 days after deposition. **D) A heat map representation of genes within the neighbourhood of *Bgdnmt1* and *Bgmbd2/3* that are significantly over or under-expressed in OVO (ovotestes).** The genes are clustered in two directions i.e. across samples and across genes. Uniprot assigned short names to these genes based on sequence homology (full name included in S2 Table) are indicated.

By taking advantage of RNA-Seq datasets generated as part of the *B*. *glabrata* genome project (Genome Publication, under review), we were able to examine the transcript abundance of the snail’s DNA methylation machinery across a range of twelve distinctive tissues (albumen gland, buccal mass, central nervous system, digestive gland/hepatopancreas, head/foot, heart/APO, kidney, mantle edge, ovotestes, salivary glands, stomach and terminal genitalia). For the purposes of examining DNA methylation machinery expression between gonadal vs. somatic tissues, samples 1 to 10 (albumen gland, buccal mass, central nervous system, digestive gland/hepatopancreas, head/foot, heart/APO, kidney, mantle edge, salivary glands and stomach) were treated as one population (Group 1), sample 11 (ovotestes) was regarded as a second population (Group 2) and sample 12 (terminal genitalia) was considered as a third population (Group 3) (Fig 5).

Differential analyses of *Bgmbd2/3*, *Bgdnmt1* and *Bgdnmt2* transcription amongst snail tissues (Group 2 vs. Group 1 or Group 3 vs. Group 1) revealed statistically significant (*p* < 0.05) increased expression of *Bgmbd2/3* in both ovotestes and terminal genitalia, *Bgdnmt1* in ovotestes and *Bgdnmt2* in terminal genitalia (Fig 5A and S1 Fig). These results were subsequently confirmed by qRT-PCR (Fig 5B). Tissue-enriched expression of *Bgdnmt1*, *Bgdnmt2* and *Bgmbd2/3* genes in gonadal structures (compared to the somatic ones) is consistent with the observations of Riviere *et al*. who demonstrated elevated transcript abundance of DNMT1, DNMT2 and MBD orthologues in *C*. *gigas* oocytes (compared to other tissues) [67]. These data collectively suggested a prominent role for these core epigenetic machinery components in molluscan gonadal tissues and cells derived from or populating them. Significant inhibition of *B*. *glabrata* egg production/embryo development, mediated by the DNA demethylating agent 5-azacytidine (5-AzaC) (Fig 5C), further supported these transcriptional results and confirmed a physiological role for DNA methylation in snail reproductive processes.

In addition to these 12 distinct tissues, *Bgdnmt1*, *Bgdnmt2* and *Bgmbd2/3* mRNA abundance was also measured by qRT-PCR in haemocytes derived from haemolymph (Fig 5B). As circulating defense cells, haemocytes are part of the snail’s innate immune system and, therefore, are involved in the host’s immune response to parasite infection [78]. Several studies have previously demonstrated that snail stress-response genes (e.g. heat shock proteins) are significantly modulated following trematode infection [79,80]. DNA methylation is commonly linked with transcriptional regulation during stress responses in eukaryotes [81,82], and indeed Ittiprasert et al. [83] have recently shown that this epigenetic modification plays a significant role during schistosome infections via the modulation of heat shock proteins. Therefore, elevated expression of the core *B*. *glabrata* DNA methylation machinery in haemocytes suggests an epigenetic link to hsp70 transcription and possibly host defense mechanisms.

Since our data support the presence of a functional *B*. *glabrata* methylation machinery, we expected to identify additional epigenetic-associated genes to be co-expressed with *Bgdnmt1*, *Bgdnmt2* and *Bgmbd2/3* in the twelve tissues analysed. Therefore, using DESeq2 [51], a pairwise differential expression analysis was performed between Group 2 (ovotestes) vs. Group 1 (somatic tissues) and Group 3 (terminal genitalia) vs. Group 1 samples to identify *Bgdnmt1*, *Bgdnmt2* and *Bgmbd2/3* co-regulated genes. Using a FDR cut-off of 10% [46] and an absolute log2 fold change of at least 1 in either of the two comparisons, over 1000 genes were significantly over- and 180 genes were significantly under- represented in ovotestes, while 850 genes were significantly over and 440 genes were significantly under- represented in terminal genitalia. Both *Bgdnmt1* and *Bgmbd2/3* passed these stringent FDR and log fold change criteria (confirming the t-distribution analysis in Fig 5A) in ovotestes (Group 2 vs. Group 1), but not in terminal genitalia. In contrast, when applying the same stringent FDR and log fold change cut-offs, *Bgdnmt2* did not display significant differential expression in either tissue.

Gene network analyses were performed to further classify the differentially expressed genes that share biological functions and similar tissue-associated transcript abundances to *Bgdnmt1* and *Bgmbd2/3*. Since the transcripts of only two of the DNA methylation machinery components (*Bgdnmt1* and *Bgmbd2/3*) were significantly up-regulated in gonadal (OVO) vs. somatic tissues, subsequent gene-network relational analyses only concentrated on these two genes. GO terms of the 1180 identified ovotestes transcripts were assigned and the relationships between these gene-products were then depicted in the form of a network of positively (R ≥ 0.6) or negatively (R ≤ -0.6) correlated genes sharing ‘Biological Process’ GO terms. Using the analogy of ‘guilt by association’ suggested by Merico and colleagues [49], the neighbourhood of *Bgdnmt1* and *Bgmbd2/3* showed a highly interconnected cluster of 53 genes (S2 Fig and S2 Table) and the expression of these genes across all 12 tissues is illustrated in the heat map in Fig 5C. Not surprisingly, the list includes genes that have been previously associated with epigenetic mechanisms or chromatin remodeling and are known for their interaction with DNMT1 homologs. For instance RBL1, a protein involved in transcriptional repression via the formation of heterochromatin by stabilising histone methylation has also a recognised function in DNMT1 transcript regulation [84]. Additionally to RBL1, the network illustrated in S2 Fig also indicates a strong link of *Bgdnmt1* with histone methyltransferases (HMT), namely SUV39H2, SETD8 and SETDB1. These findings are in line with studies reported for mammalian HMTs, which are known to associate with or modulate DNA methyltransferases [85,86].

### The *B*. *glabrata* DNA methylation machinery is differentially active in inbred snail strains vs. outbred hybrids

**Fig 6:**
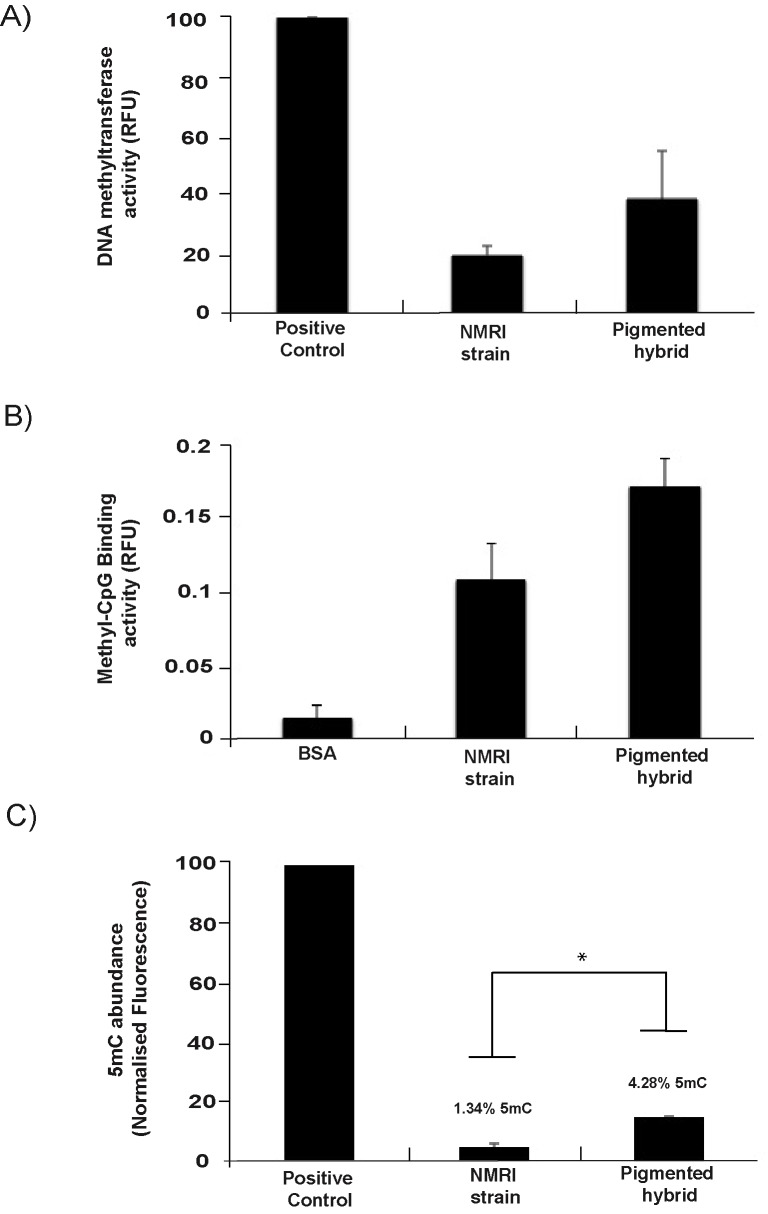
Outbred (pigmented) *B*. *glabrata* snails contain higher DNA methyltransferase activity, MBD-binding capacity and genome 5mC compared to inbred (NMRI) snails. DNA methyltransferase and MBD-binding activity was fluorometrically measured in nuclear protein isolated from the head/foot tissue of adult snails. **A) DNMT activity was measured in 7** μ**g of *B*. *glabrata* (NMRI and pigmented hybrid strains) nuclear protein extract (n = 2) using EpiQuik DNA Methyltransferase Activity/Inhibition Assay Kit (Epigentek).** Relative fluorescence units (RFU) were obtained at 530_EX_/590_EM_ nm and subsequently normalised to the blank negative control (assay buffer only) and positive control (Dnmt1). Error bars represent standard deviation (SD) of the normalised means. **B) MBD-binding activity within 10** μ**g of *B*. *glabrata* (NMRI and pigmented hybrid strains) nuclear protein extract (n = 2) was measured with the EpiQuik MBD2 Binding Activity/Inhibition Assay Kit (Epigentek).** 10 μg of BSA was used as a negative control. Fluorescence was read at 530_EX_/590_EM_ nm and readings subsequently normalised to the blank negative control (assay buffer only). Error bars represent ± standard deviation (SD) of the normalised means. **C) 5mC was detected in *B*. *glabrata* gDNA (100ng) derived from both albino NMRI and pigmented hybrid strains (n = 2) using the MethylFlash methylated DNA Quantification Kit (Epigentek).** The level of 5mC was measured in relative fluorescence units (RFU) at 530_EX_/590_EM_ nm and normalised to the negative (synthetic unmethylated DNA with 50% cytosine content) and positive control (synthetic methylated DNA with 50% 5mC content). * indicates a significant difference (Student’s two-tailed t test; *p<0*.*05*) between the 5mC level of NMRI and Pigmented snails. Readings are shown as means and error bars represent ± standard deviation (SD). 5mC abundance (%), displayed above bars, was calculated based on the *B*. *glabrata* genome GC content (35%) as described in the Materials and Methods.

While a functional DNA methylation machinery has previously been reported in *B*. *glabrata*, direct comparisons of DNA methyltransferase and MBD activity between different snail populations (e.g. inbred vs. outbred individuals) are lacking. This prompted us to measure both DNA methyltransferase [87] and MBD binding activity [88] within nuclear protein extracts derived from the head/foot of adult NMRI inbred and pigmented outbred snail populations as well as to quantify m5C levels in their gDNA pools (Fig 6).

Firstly, using an ELISA-based assay, measurable amounts of DNMT activity were present in nuclear extracts of both strains (Fig 6A). This data corroborates our description of putative functional BgDNMT1 and BgDNMT2 family members (Fig 3) and confirms the observations of others [26,27,83]. Interestingly, these DNMT activity levels were elevated in the pigmented hybrid strain when compared to the NMRI inbred strain. We secondly assessed MBD binding activity (again using an ELISA-based assay) in the same samples, which revealed that the nuclear protein extracts of both snail strains additionally contain MBD proteins capable of binding to methylated DNA (supporting the bioinformatics identification of a putative functional BgMBD2/3, Fig 1). Similar to the DNMT assay, MBD activity is higher in the pigmented hybrid snail samples (Fig 6B). Finally, total 5mC levels were fluorometrically quantified within gDNA samples derived from both NMRI and pigmented *B*. *glabrata* populations (Fig 6C). Based on a genomic CG content of 35%, (Genome Publication, under review) the amount of total cytosine methylation was estimated at 1.34% and 4.28% for the NMRI and the pigmented hybrid strain respectively. These values are within the range of DNA methylation levels detected in other invertebrates [89], similar to the percentage of 5mC found in another mollusc [90] and close to the 2% previously reported by Fneich *et al*. [26] in the BgBRE strain using an LC-MS-based approach. Interestingly, the significantly higher levels (*p* < 0.05) of detectable 5mC within gDNA pools of the pigmented hybrid in comparison to the NMRI strain is in line with the MBD and DNMT activity assays (Fig 6A & 6B).

It is commonly accepted that plant and animal hybrids frequently display different traits and increased fitness in comparison to inbred populations (e.g. increased fecundity [91,92]). This boost in performance is generally referred to as hybrid vigour or heterosis, and so far, epigenetic mechanisms underlying this phenomenon have not been thoroughly characterised [93,94]. Recently, however, the role of epigenetics has been implicated with several studies demonstrating the importance of small RNA-directed DNA methylome dynamics in increasing hybrid performance (e.g. Groszmann *et al*. [95]). Additionally, and more pertinent to our current findings were those reported by Shen and colleagues, who discovered that elevated 5mC levels in hybrid individuals led to global transcriptional changes and contributed to heterosis in *Arabidopsis thaliana* [96]. While our observations could simply reflect differences in life history traits, more thorough analyses of DNA methylation in *B*. *glabrata* populations that display different susceptibilities to schistosome infection, maintain different geographical distributions or are subject to diverse laboratory pressures may shed additional light on the proposed role of this epigenetic process in molluscan heterosis.

### High resolution BS-PCR analysis reveals the presence of 5mC within exons of the *B*. *glabrata* 14-3-3 gene

**Fig 7:**
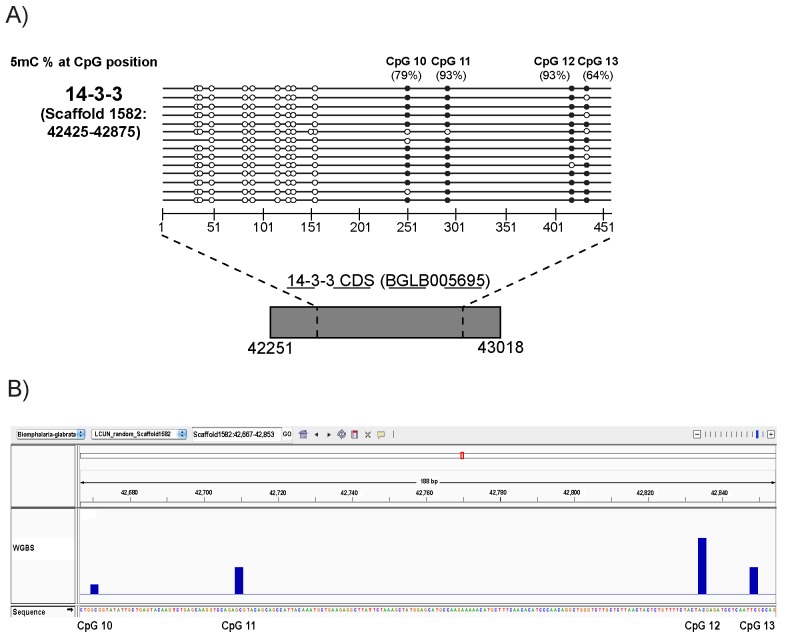
The *B*. *glabrata* 14-3-3 (BGLB005695) single exon gene contains methylated cytosines within its coding region. **A) PCR amplification of bisulfite converted DNA reveals four 5mC sites within Bg14-3-3.** Bisulfite conversion of gDNA followed by PCR (BS-PCR) and sequencing was employed to investigate the methylation status of cytosines within the exon of *Bg14-3-3* (Scaffold1582:42425–42875). Following bisulfite conversion of gDNA, target sequences were amplified, subcloned, and individual clones subsequently sequenced. A filled circle indicates the presence of 5mC; an empty circle indicates the absence of 5mC. Each circle represents a cytosine in a CpG context. Numbers above the circles correspond to base pair positions. Percentages of 5mC detected at each cytosine are indicated. Grey box represents CDS of *Bg14-3-*3 and the dashed lines indicate the amplified region. B) **Confirmation of methylated CpGs within *Bg14-3-3* by WGBS.** An IGV v2.3 [57] genome browser screenshot of the *Bg14-3-3* gene. Black bars indicate a methylated CpG position as determined by WGBS (Genome Publication, under review) and y-axis represents degree of methylation (between 0–1) as estimated by BSMAP v1.0.0 [100].

While Fneich *et al*. [26] have previously demonstrated that the non-LTR repetitive element *Nimbus* (*BgI*) is either highly methylated or unmethylated, the same authors have proposed that the *B*. *glabrata* genome consists of densely methylated regions, interspersed with stretches of unmethylated DNA, due to the bimodal distribution the CpG observed to expected ratio (CpGo/e) within protein coding genes. This so-called mosaic DNA methylation pattern was further confirmed by a Whole Genome Bisulfite-Seq (WGBS) experiment as part of the *B*. *glabrata* genome project (Genome Publication, under review). This observation is in line with numerous invertebrate studies [73,97–99] and describes the existence of two types of methylated genes, those that are highly methylated (coding for house-keeping proteins) and those that are lowly methylated (encoding inducible gene products). Therefore, to support the WGBS analysis of the snail’s genome and to confirm the *in silico* CpG*o/e* predictions of Fneich *et al*. [26] as well as to maximise our chances at identifying robust 5mC signals within a *B*. *glabrata* protein coding gene (similar to that recently detected for Bg-hsp70 [83], we analysed the methylation status of a 451 bp region of the house-keeping *Bg14-3-3* gene (Scaffold 1582:42425–42875) (Fig 7A).

14-3-3 genes code for highly conserved proteins ubiquitously expressed in eukaryotes and due to their interaction with signalling molecules, are involved in various biological pathways [101]. By analysing 14 sub-cloned BS-PCR amplicons of *Bg14-3-3* and assessing the methylation status of 13 CpG sites contained within this single exon gene, we were able to confirm the CpGo/e prediction and WGBS detection of DNA methylation within the exonic region (451bp) of this housekeeping *B*. *glabrata* gene. Specifically, ~30% of the total CpG sites within this region of *Bg14-3-3* contain a methylation mark, and four CpG positions are methylated across nearly all clones (Fig 7A). The DNA methylation status of these four CpG sites (CpG10-CpG13) was also conserved in the WGBS data set (Genome Publication, under review) (Fig 7B), confirming the stability of these epigenetic marks within this specific locus. Intragenic (gene-body) methylation has been positively linked to transcription [74,89,102]. Hence, congruent with other invertebrate species, [99,103,104] and supported by both WGBS and *in silico* analyses of the *B*. *glabrata* genome [26], snail DNA methylation appears predominantly directed towards transcriptional units of house-keeping function (e.g. 14-3-3 in the current study). The mammalian 14-3-3 homolog is known to be regulated by epigenetic modifications and aberrant DNA methylation patterns have been linked to tumourgenesis [105]. Relevantly, we were able to demonstrate 5mC within an exonic region of *Bg14-3-3* and hence propose a similar regulatory role of DNA methylation for the *B*. *glabrata* homolog as well.

Additionally, in contrast to some organisms, such as *D*. *melanogaster* [76], *Dictyostelium discoideum* [65] and *Entamoeba histolytica* [106], where non-CpG (i.e. CpH (H = T, A or C)) methylation is frequently observed, but in common with other molluscs (e.g. *C*. *gigas*; [107] and *Chlamys farreri* [25]), DNA methylation in *B*. *glabrata* appears to be generally restricted to a CpG context (all cytosines in a non-CpG context were converted after bisulfite treatment). These findings are in line with recent observations by Ittipraset *et al*. [83] and the recently reported *B*. *glabrata* genome paper (Genome Publication, under review). As it is generally believed that genomes containing a Dnmt1 homolog mainly display methylation within CpG dinucleotides [71,89], our results (*B*. *glabrata* contains a DNMT1 homolog, Fig 3 and harbours CpG methylation, Fig 7A & 7B) are in line with this view.

## Schistosome products modulate the transcription of *Bgdnmt1* and *Bgmbd2/3*

**Fig 8:**
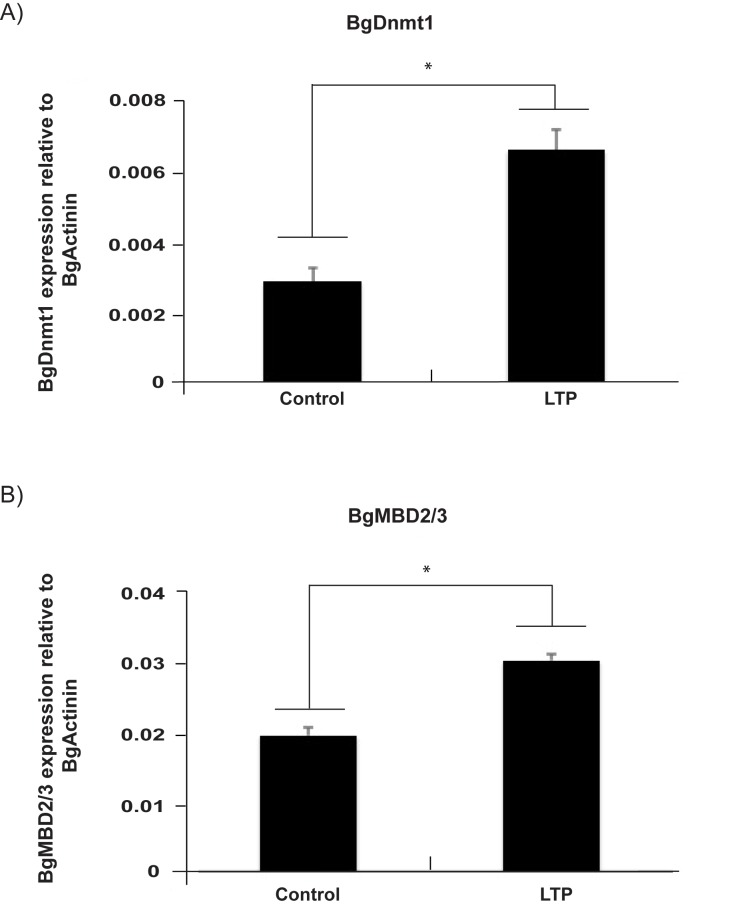
*S*. *mansoni* parasite products significantly increases transcript abundance of core *B*. *glabrata* DNA methylation machinery components. qRT-PCR expression analysis of: **A)** *Bgmbd2/3* and **B)** *Bgdnmt1* in untreated *B*. *glabrata* embryonic (Bge) cells (control) versus cells treated with Larval Transformation Products (LTP) [58]. Data is derived from duplicate biological samples and qRT-PCR reactions were performed in technical duplicates. The Ct-values of target genes were normalised to *Bgactin* [116]. Error bars represent standard deviation (SD) of the normalised means and a Student’s t-test was performed to identify expression differences between LTP-treated and untreated (Control) Bge cells (*; *p < 0*.*05*).

To successfully parasitise the molluscan intermediate host, schistosomes have to overcome the snail’s immune response. While the exact mechanisms by which schistosome parasites accomplish this feat are incompletely understood, the Bge cell line provides a powerful *in vitro* culture model to investigate the complex host-parasite interplay [108,109]. Bge incubation with larval transformation products (LTP) derived from miracidia to sporocyst transformation is thought to mimic the events that normally occurs inside the molluscan host [110]. Several studies have previously demonstrated that parasite-mediated modulation of various snail genes occurs [77,111,112] with Knight *et al*. [113] further demonstrating that gene repositioning within the snail nucleus occurs post parasite exposure. These nuclear reorganisation events, which are non-random, are known to impact gene expression and can be trigged by the presence of methylated CpGs [114,115]. Here, to explore whether schistosome products impact the transcriptional regulation of snail DNA methylation machinery components, Bge cells were cultured in the presence or absence of schistosome LTP [58] and assessed for *Bgdnmt1* and *Bgmbd2/3* abundance (Fig 8).

Interestingly, Bge cells exposed to schistosome LTP significantly increased their expression of both *Bgdnmt1* (Fig 8A) as well as *Bgmbd2/3* (Fig 8B) indicating that the snail’s epigenetic machinery is responsive to biotic stress and is specifically reactive to parasite products. While translation of our data from a cellular system to whole organisms must be cautiously tempered, a recent study demonstrated that tissue-specific DNA methylation of snail *Bg-hsp-70* is temporally affected by natural schistosome exposure and infection [83]. Collectively, these data would support the plasticity of the schistosome-modulated *B*. *glabrata* DNA methylation machinery in both cell (Bge) and whole organism (snails) systems. To further explore the functional relevance of DNA methylation-mediated processes during the snail’s response to parasite infection and to identify which specific pathways are epigenetically modulated, genome-wide DNA methylation/transcriptome analysis of infected vs. non-infected individuals (or cells derived from them) should be considered.

## Conclusions

The increasing risk of *S*. *mansoni* transmission due to territory extension of its molluscan host *B*. *glabrata* poses a great concern even for developed countries in temperate regions. Since current mass drug administration programmes have limitations [15,16] and past intermediate host eradication programmes were largely unsuccessful [14,117], the development of novel lifecycle intervention strategies is instrumental for the future control of schistosomiasis. Using a multidisciplinary approach, this study comprehensively characterised the core DNA methylation machinery of a gastropod mollusc as well as illustrated that it is more abundantly expressed in gonadal vs. somatic tissues, is differentially active in hybrid vs. inbred snail populations and is responsive to schistosome soluble products. This extended knowledge of *B*. *glabrata* epigenetics importantly provides new targets and molecular processes that could be instrumental in the development of integrated ways to combat a major neglected tropical disease.

## Supporting information

S1 Table Primer sequences. (XLSX)

S2 Table Uniprot IDs and names of genes within neighbourhood of *Bgdnmt1* and *Bgmbd2/3*. (CSV)

S1 Fig *Bgdnmt1*, *Bgdnmt2* and *Bgmbd2/3* RNA-Seq data (Fig 5A) superimposed onto the eleven *B*. *glabrata* tissues analysed. Colour shades correspond to standardised RNA-Seq counts.
(PDF)

S2 Fig *Bgmbd2/3* and *Bgdnmt1* network analysis. Interconnected cluster of genes significantly overexpressed (green circles) or underexpressed (grey circles) in OVO and within the neighbourhood of *Bgmbd2/3* and *Bgdnmt1*. Each gene is depicted by a vertex (node) and two adjoining genes are referred to as neighbours if they are connected by a line (edge). Abbreviations refer to UniProt gene IDs and full names are listed in S2 Table. BgDNMT1 and BgMBD2/3 are indicated by red vertexes and are connected by a red edge.
(PDF)
