# Supplementary figures and images for: The Biomphalaria glabrata DNA methylation machinery displays spatial tissue expression, is differentially active in distinct snail populations and is modulated by interactions with Schistosoma mansoni
Source: PLoS Negl Trop Dis. 2017 May 16;11(5):e0005246. doi: 10.1371/journal.pntd.0005246 (PMC5433704; doi:10.1371/journal.pntd.0005246)

Bgdnmt1

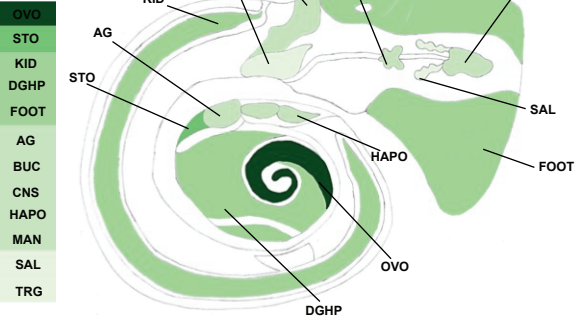

Bgdnmt2

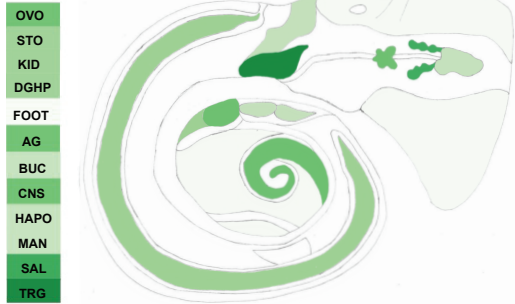

Bgmbd2/3

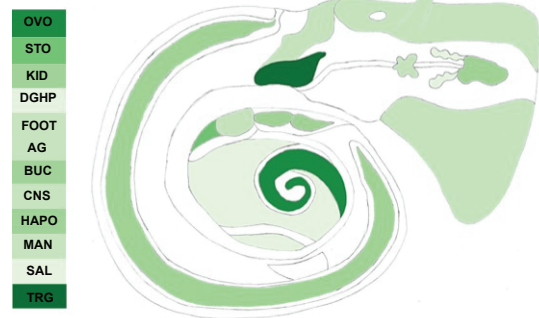

Standardised counts

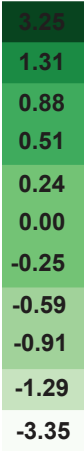

Supplement: S1 Fig — Colour shades correspond to standardised RNA-Seq counts. (PDF) [file pntd.0005246.s003.pdf]

# BgDnmt1 & BgMBD2/3 Neighbours

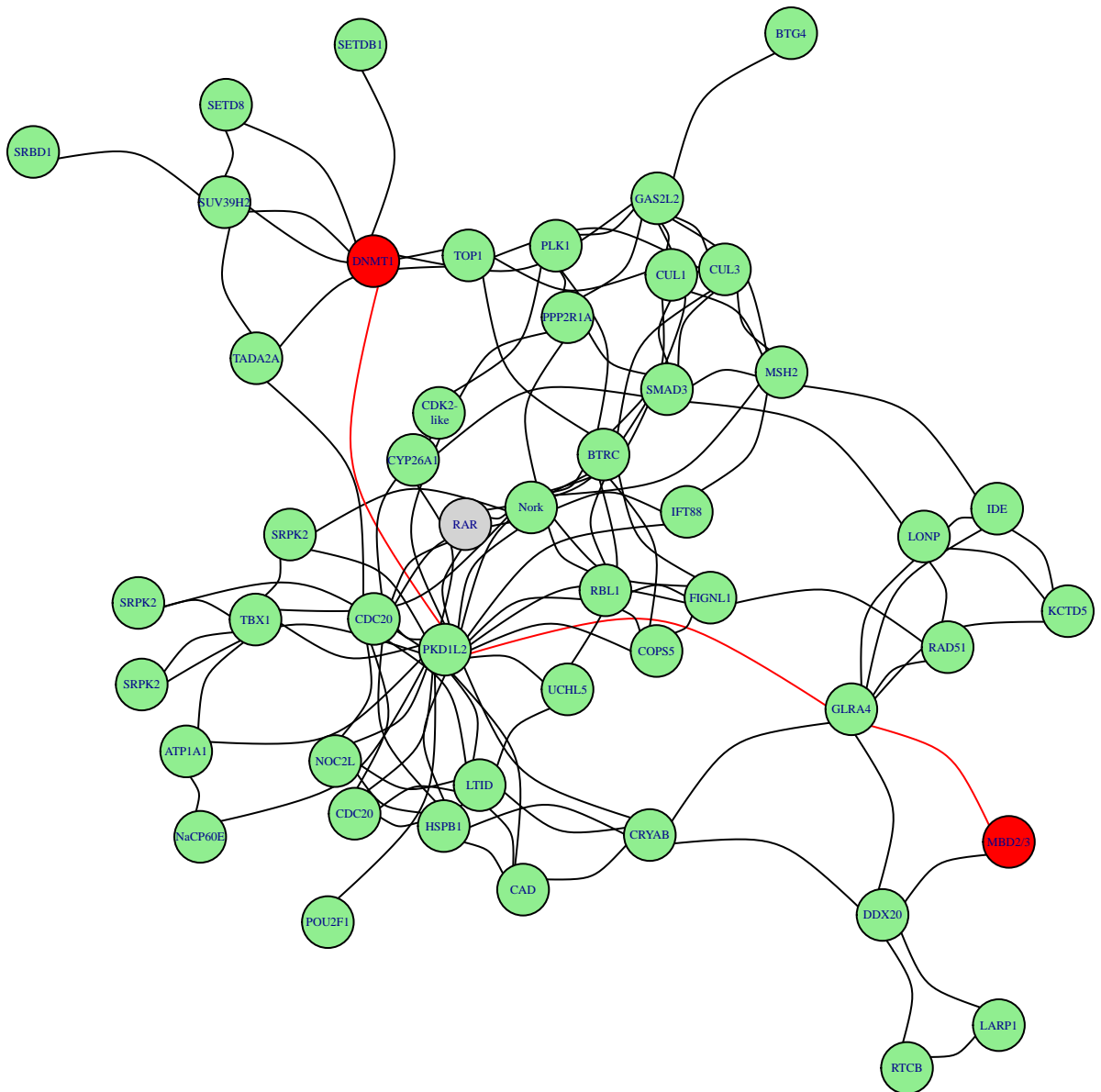

Supplement: S2 Fig — Interconnected cluster of genes significantly overexpressed (green circles) or underexpressed (grey circles) in OVO and within the neighbourhood of Bgmbd2/3 and Bgdnmt1. Each gene is depicted by a vertex (node) and two adjoining genes are referred to as neighbours if they are connected by a line (edge). Abbreviations refer to UniProt gene IDs and full names are listed in S2 Table. BgDNMT1 and BgMBD2/3 are indicated by red vertexes and are connected by a red edge. (PDF) [file pntd.0005246.s004.pdf]
